# Expression Dynamics and Estrogen Response of Estrogen Receptors in Duolang Sheep During Puberty

**DOI:** 10.3390/genes16070731

**Published:** 2025-06-24

**Authors:** Lexiao Zhu, Gul Muhammad Shahbaz, Huiping Sun, Jihu Zhang, Wei Li, Ruohuai Gu, Feng Xing

**Affiliations:** 1 College of Animal Science, Tarim University, Alar 843300, China; 18538512047@163.com (L.Z.); shahbazbalouch42@gmail.com (G.M.S.); sunhuiping223@163.com (H.S.); 13392140210@136.com (J.Z.); 18597814328@163.com (W.L.); 17591551047@163.com (R.G.); 2 Key Laboratory of Tarim, Animal Husbandry Science and Technology, Xinjiang Production & Construction Corps, Alar 843300, China

**Keywords:** Ovisaries, estrogen, ERs, Duolang sheep, puberty, granulosa cells

## Abstract

Background/Objectives: Puberty is a critical stage in sheep development when reproductive capability is established, but the hormonal mechanisms underlying this transition remain incompletely understood. This study aimed to investigate the dynamic changes in estradiol (E2) levels and the expression patterns of estrogen receptors (ERα and ERβ) during puberty in Duolang sheep, a breed characterized by early sexual maturity and high reproductive efficiency. Methods: A total of 18 female Duolang sheep were assigned to three developmental stages (*n* = 6 per group): prepuberty (145 days), puberty (within 0 h of first estrus), and postpuberty (+3 days). Serum E2 concentrations and the mRNA and protein levels of ERα and ERβ were assessed in the hypothalamus, pituitary, and ovary. Additionally, primary ovarian granulosa cells (GCs) were isolated and stimulated in vitro with increasing concentrations of E2 (0–1000 ng/mL) to evaluate the dose-dependent expression of ERα, ERβ, and gonadotropin-releasing hormone (GnRH). Results: E2 levels peaked at the onset of puberty and declined thereafter. ERα expression in the hypothalamus and pituitary decreased during puberty but rebounded postpuberty, indicating a role in negative feedback regulation. In contrast, ovarian ERα expression reached its highest level during puberty, while ERβ expression in the ovary gradually increased from prepuberty to postpuberty. In GCs, ERα exhibited a biphasic expression pattern, peaking at 250 ng/mL E2 and decreasing at higher concentrations. ERβ and GnRH expression levels increased in a dose-dependent manner. Conclusions: These findings suggest that ERα primarily mediates E2 feedback within the hypothalamus–pituitary axis, whereas ERβ is associated with ovarian development and may regulate GnRH expression during the pubertal transition. The study provides new insights into the hormonal regulation of puberty in Duolang sheep and offers potential biomarkers for improving reproductive efficiency through targeted breeding strategies.

## 1. Introduction

Puberty in animals marks the onset of reproductive capability, characterized by the first estrus and ovulation [1]. The timing of puberty in mammals is significantly influenced by genetic factors [2]. The hypothalamus–pituitary–ovary (HPO) axis is a crucial regulatory pathway in the neuroendocrine system, with puberty representing the activation of this axis [3]. Gonadotropin-releasing hormone (GnRH) is secreted from the hypothalamus to the anterior pituitary in a pulsatile manner during puberty, stimulating the synthesis and secretion of follicle-stimulating hormone (FSH) and luteinizing hormone (LH) [4]. FSH and LH circulate to the ovaries, promoting their development and estrogen synthesis [5].

Estrogen, particularly 17β-estradiol (E2), is the most biologically active form [6] and binds to two estrogen receptors (ERα and ERβ) encoded by the *ESR1* and *ESR2* genes [7]. E2 specifically binds to these receptors, stimulating the proliferation of ovarian GCs and enhancing their response to gonadotropins [8]. Both ERs are members of the nuclear receptor family [9] and regulate gene transcription in response to E2 [10]. Previous studies have detected ERα and ERβ in the hypothalamus and pituitary of sheep [11] indicating their significant role in reproductive regulation. Duolang sheep, a representative Xinjiang breed [12], exhibit early puberty and year-round estrus, with first estrus occurring at approximately 145–150 days of age (≈4.8–5.0 months) [13]. This is notably earlier than the average age at first estrus in other Chinese breeds such as Small Tail Han sheep (~180 days) and Western breeds including Dorset (~200 days) and Suffolk (~220 days). Such early sexual maturity makes Duolang sheep a valuable model for elucidating mechanisms underlying puberty onset. This study aims to elucidate the changes in E2 and ER expression during the onset of puberty in Duolang sheep, as well as the effects of E2 on ER expression in ovarian granulosa cells (GCs).

## 2. Materials and Methods

### 2.1. Animal Care

This study adhered to the ethical guidelines set by the Ethics Committee of Tarim University of Science and Technology (approval number SYXK 2020-009), approved on 23 April 2020.

### 2.2. Animals and Tissue Collection

Eighteen healthy female Duolang sheep were selected and categorized into three groups: prepuberty (145 days of age), puberty (within 4–6 h after the onset of first estrus), and postpuberty (3 days after estrus), with six animals per group (*n* = 6). Estrus was monitored twice daily (08:00 and 18:00) using a teaser ram, combined with behavioral signs including tail fanning, standing reflex, vulvar swelling, and mucus discharge. Once estrus was detected, animals were monitored every 2 h to accurately define the pubertal time point and capture peak E2 levels. Sheep were anesthetized with 3% pentobarbital sodium (1 mL/kg, i.v.) before tissue collection. The hypothalamus, pituitary, and ovaries were harvested, and ovarian morphology was recorded. All animal handling, anesthesia, and tissue collection procedures were performed by or under the supervision of a licensed veterinarian, in accordance with institutional animal care guidelines. All samples were snap-frozen in liquid nitrogen and stored at −80 °C.

### 2.3. Blood Sample Collection and Hormone Measurement

To assess dynamic changes in serum estradiol (E2) levels during puberty, six Duolang ewes were continuously monitored. Beginning at the onset of first estrus, blood samples were collected from the same animals every 2 h over a 72 h period via jugular venipuncture (2.5 mL per sample). All sampling was performed by experienced handlers using gentle and consistent techniques to minimize stress. Blood sampling was performed by trained veterinary staff to ensure animal welfare and sampling accuracy. No abnormal behaviors or disruptions in estrous expression were observed during the sampling period, indicating that repeated blood collection did not interfere with physiological status. Samples were centrifuged at 1000× *g* for 15 min at 4 °C, and serum was aliquoted and stored at −20 °C until analysis. E2 concentrations were determined using a commercially available ELISA kit (Beijing Huaying Institute of Biotechnology, Beijing, China), following the manufacturer’s instructions. The intra-assay and inter-assay coefficients of variation were <10%. All serum samples were assayed in duplicate on a single ELISA plate to eliminate inter-plate variability. Serum estradiol (E2) concentrations were measured using a commercial ELISA kit specific for sheep (Jiangsu Jingmei Biotechnology Co., Ltd., Yancheng, Jiangsu, China). According to the manufacturer’s instructions, the intra-assay and inter-assay coefficients of variation (CVs) were <10% and <12%, respectively. The assay sensitivity (lower limit of detection) was 3 pg/mL.

### 2.4. Granulosa Cell Culture

Fresh DL ovaries were rinsed with 75% alcohol and PBS. Follicular fluid was extracted and mixed with a DMEM culture medium. The mixture was centrifuged, and GCs were cultured in a complete DMEM medium at 37 °C with 5% CO_2_ for 24 h before stimulation with different E2 concentrations (Beyotime, Shanghai, China).

Ovaries were collected from pubertal Duolang ewes. Early antral follicles (2–5 mm) were aspirated, and the follicular fluid was filtered through a 70 μm mesh to remove oocytes and debris. The cell suspension was centrifuged at 1500× *g* for 5 min and resuspended in DMEM with 10% FBS and 1% penicillin–streptomycin. Cells were seeded into T25 flasks or 24-well plate and cultured at 37 °C with 5% CO_2_. Medium was changed every 48 h. All experiments were performed using passage 1 cells.

### 2.5. Granulosa Cell Identification

Granulosa cell identity was confirmed by immunofluorescence staining for follicle-stimulating hormone receptor (FSHR). Cells were seeded on sterile glass coverslips in 24-well plates (0.02 × 10^6^ cells/well) and cultured for 2 h or overnight. After fixation with 4% paraformaldehyde (PFA, Solarbio Life Sciences, Beijing, China), cells were permeabilized and blocked with 0.5% Triton X-100 and 10% goat serum. Cells were incubated with primary anti-FSHR antibody (1:100, Proteintech Group, Inc., Wuhan, China) overnight at 4 °C, followed by Alexa Fluor 488–conjugated secondary antibody (1:500, Proteintech Group, Inc., Wuhan, China) for 2 h at room temperature. Nuclei were stained with DAPI (1:1000, Solarbio Life Sciences, Beijing, China), and coverslips were mounted with Fluoromount-G (SouthernBiotech, Birmingham, AL, USA). Images were acquired at 200× magnification.

### 2.6. Total RNA Isolation and cDNA Synthesis

RNA was extracted using Trizol (Invitrogen, Carlsbad, CA, USA), and cDNA was synthesized using the PrimeScript RT reagent Kit (TaKaRa Bio Inc., Dalian, China). According to the instructions, amplification was carried out in a reaction system of 20 μL. The reaction was incubated for 50 min at 42 °C, followed by 5 min at 95 °C to inactivate the reverse transcriptase.

### 2.7. Quantitative Real-Time PCR and Cloning

Sequences of sheep ERs were found from Gene Bank, and primers were designed according to Primer Premier 6.0. Using the cDNA of Duolang sheep as a template, the expression levels of ERs in tissues were detected by PerfectStart Green qPCR SuperMix (Trans, Beijing, China). The total reaction system was 15 μL. qPCR was performed under the following conditions: 95 °C for 10 min, followed by 40 cycles at 95 °C for 20 s and 60 °C for 30 s, then at 95 °C for 15 s, 60 °C for 30 s, and 95 °C for 15 s. Each sample was run three times.

ERs were cloned by reverse transcriptase-PCR (RT-PCR). The total RT-PCR reaction system was 25 μL, and each system contained 1 μL cDNA. The reaction was incubated at 95 °C for 5 min, followed by 35 cycles of 95 °C for 30 s, annealing for 30 s and 72 °C for 1 min, followed by one cycle of 72 °C for 5 min. The RT-PCR product connected with PMD-19T (TaKaRa Bio Inc., Dalian, China). The well-reacted ligands were mixed with DH5α (Trans, Beijing, China) receptor cells. The recombinants were selected by blue-white spot reagent and amplified by PCR. The qualified bacterial solution was sent for sequencing. Amplification specificity was confirmed by melt curve analysis and 1.5% agarose gel electrophoresis, which showed single bands of the expected size. The sequences of primers and product lengths are listed in Table 1.

### 2.8. Western Blot Analysis of ER Expression

Hypothalamus, pituitary, and ovary samples at different stages were thoroughly ground at low temperatures. Protein lysis buffer was added, and samples were lysed fully on ice. Lysates were centrifuged at 1000× *g* for 15 min at 4 °C, and the supernatants were collected and denatured in a 98 °C water bath. Samples were stored at −20 °C until use. For SDS-PAGE electrophoresis, 8 μL of total protein per sample was loaded onto each well. After electrophoresis, the corresponding bands for ERα (66 kDa), ERβ (59 kDa), and GAPDH (36 kDa) were excised, and proteins were transferred onto PVDF membranes. The membranes were blocked and incubated overnight at 4 °C with the following primary antibodies (all from Proteintech, Wuhan, China): anti-ERα (Cat# 21244-1-AP, 1:1000), anti-ERβ (Cat# 14007-1-AP, 1:2000), and anti-GAPDH (Cat# 10494-1-AP, 1:10,000). After washing three times (10 min each), membranes were incubated with HRP-conjugated secondary antibody (goat anti-rabbit IgG, Cat# SA00001-2, 1:2000, Proteintech) at 37 °C for 1 h. The membranes were then washed three times for 10 min each. Protein bands were visualized using an enhanced chemiluminescence detection kit (Millipore, Billerica, MA, USA). Each group included three biological replicates (*n* = 3). Due to membrane size constraints, Western blots for different stages were performed on separate membranes; however, all were run under identical conditions using the same batch of reagents and processed simultaneously. Band intensities were normalized to β-actin and quantified using ImageJ software (version 1.53a, National Institutes of Health, Bethesda, MD, USA).

### 2.9. Statistical Analysis

All data were analyzed using SPSS version 24.0 (IBM, Armonk, NY, USA). Prior to statistical testing, data were assessed for normality using the Shapiro–Wilk test and for homogeneity of variances using Levene’s test. One-way analysis of variance (ANOVA) was performed to evaluate differences among groups, followed by Tukey’s post hoc test for multiple comparisons. Results are presented as the mean ± standard error of the mean (SEM), and differences were considered statistically significant at *p* < 0.05. Although a formal power analysis was not conducted, a sample size of *n* = 6 per group was consistent with previous studies in reproductive endocrinology of sheep and was considered sufficient to detect biologically meaningful differences.

## 3. Results

### 3.1. Changes in Estrogen Levels

E2 levels in Duolang sheep during puberty showed a pulsatile pattern, with two prominent peaks at 0 h and 34 h. Levels were significantly lower during the prepubertal stage and increased during puberty, followed by a sharp decline in the postpubertal stage.

**Figure 1:**
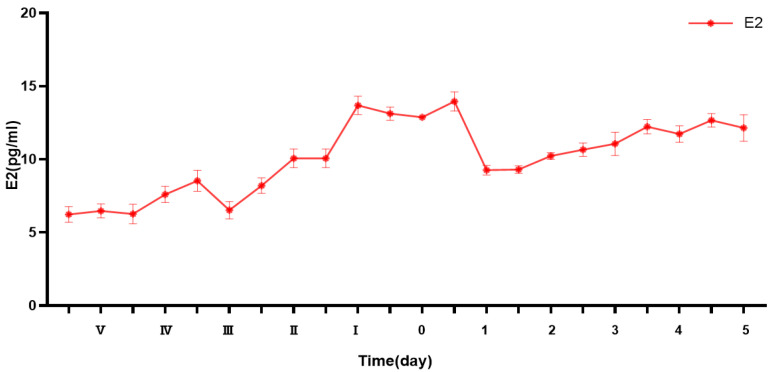
Represents the day when Duolang sheep accepted mounting. E2 level at the onset of puberty. V, IV, III, II, and I represent five, four, three, two, and one days before 0.

E2 secretion showed a pulsatile pattern during the pubertal transition in Duolang sheep (Figure 1). E2 levels were relatively low during the prepubertal stage, and then increased significantly and peaked near the onset of puberty. During the late pubertal period, E2 levels declined sharply, followed by a gradual increase in the postpubertal stage, reaching levels higher than those observed in prepuberty.

**Figure 2:**
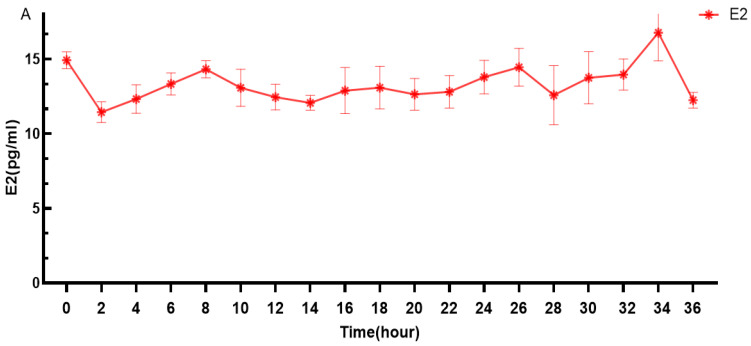

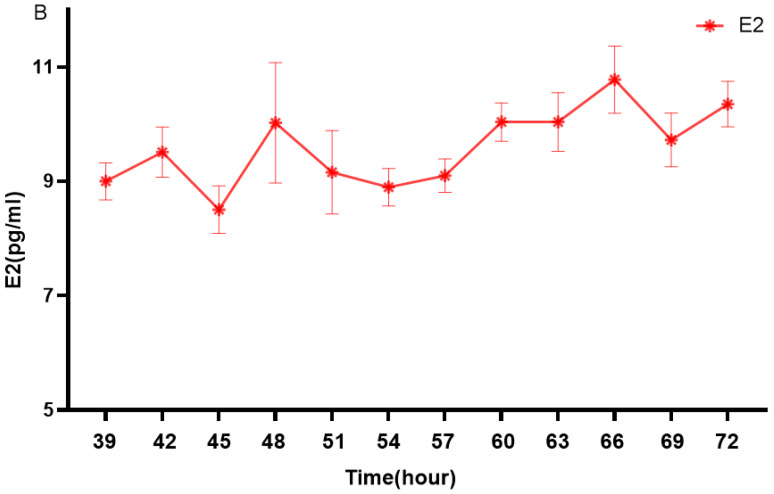
(**A**,**B**) Serum estradiol (E2) levels in Duolang sheep during the 72 h period surrounding the onset of puberty. “0 h” represents the estimated time point of first estrus, which is considered the onset of puberty. The corresponding pubertal stages—prepuberty (−72 to 0 h), puberty (0 h), and postpuberty (0 to +72 h).

As shown in Figure 2, E2 of Duolang sheep in puberty was pulsated within 0–36 h. At 0 h (14.917 ± 0.563 pg/mL) and 34 h (16.765 ± 0.893 pg/mL), two obvious peaks appeared (Figure 2A). At 2 h (11.438 ± 0.689 pg/mL), E2 level was low. Then, there was a small fluctuation until 32 h (13.956 ± 1.037 pg/mL). The E2 level reached its peak at 34 h (16.765 ± 0.893 pg/mL). It can be seen (Figure 2B) that after 34 h, a rapid decline followed.

### 3.2. Ovarian Morphology Observations

**Figure 3:**
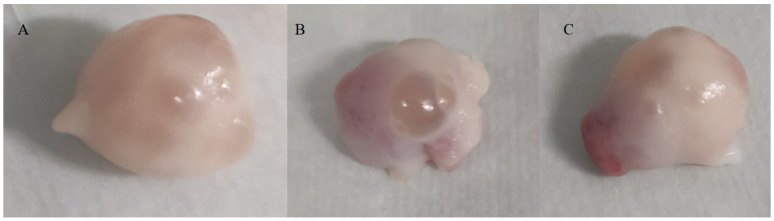
Ovaries of Duolang sheep at different stages. (**A**) was in prepuberty, (**B**) was in puberty, and (**C**) was in postpuberty.

Ovaries at puberty displayed smaller, immature follicles, while larger follicles were evident. Postpuberty, the absence of estrus symptoms, and the presence of a corpus luteum were noted. Several smaller and immature follicles were observed in the ovaries at puberty (Figure 3A). A large follicle (Figure 3B) with thin walls protruding from the surface of the ovary was evident in puberty. In postpuberty, the estrus symptoms disappeared. And the follicle wall ruptured, the egg was expelled from the follicle, forming a red body (Figure 3C).

### 3.3. Identification of Primary Granulosa Cells

**Figure 4:**
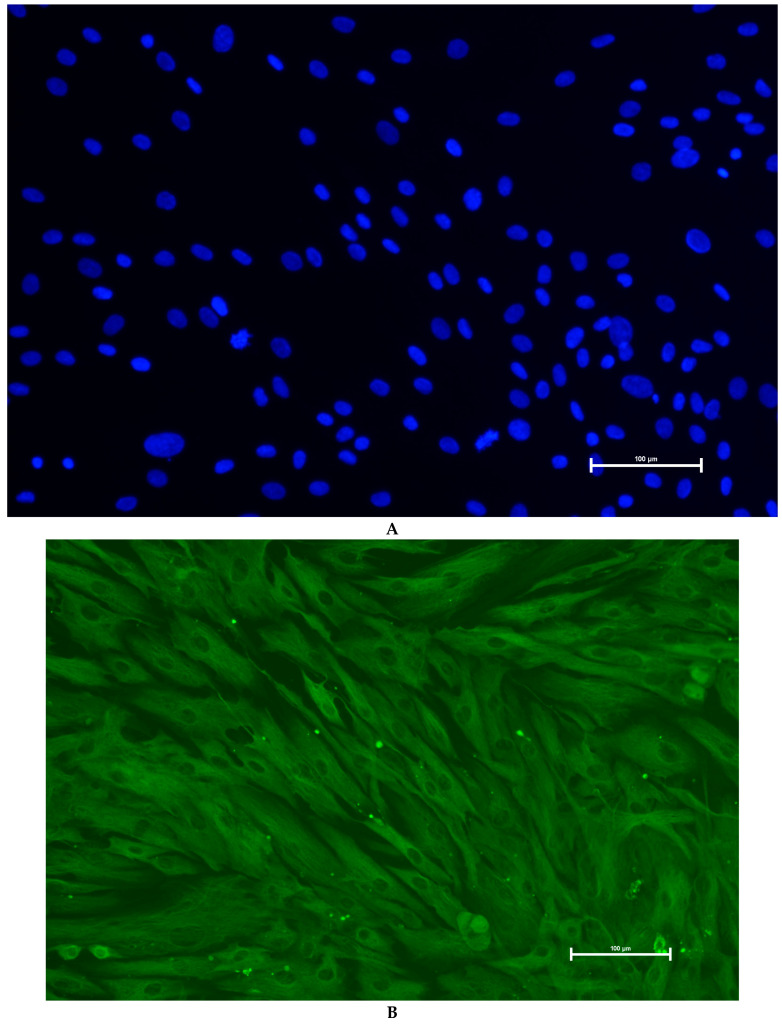

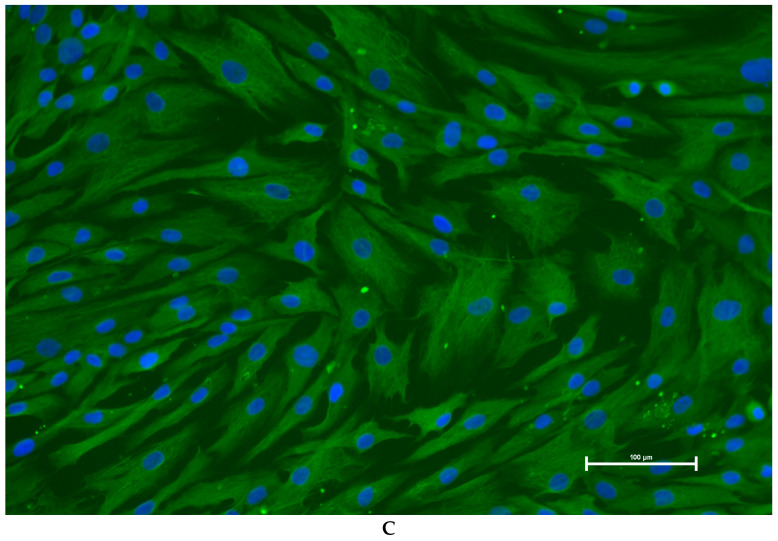
Immunofluorescence staining of granulosa cells. (**A**) DAPI-stained nuclei (blue); (**B**) FSHR-specific signal (green); (**C**) merged image. Positive cytoplasmic FSHR confirms granulosa cell identity. Magnification: 200×.

To verify the identity of isolated granulosa cells, immunofluorescence staining for FSHR was performed. As shown (Figure 4), the cells exhibited characteristic cobblestone morphology under phase-contrast microscopy and showed strong cytoplasmic FSHR expression. DAPI staining confirmed nuclear integrity, and merged images demonstrated clear colocalization. These results confirmed that the cultured cells were granulosa cells with high purity.

### 3.4. Expression of ERs and PGR in Different Tissues and Periods of Duolang Sheep

**Figure 5:**
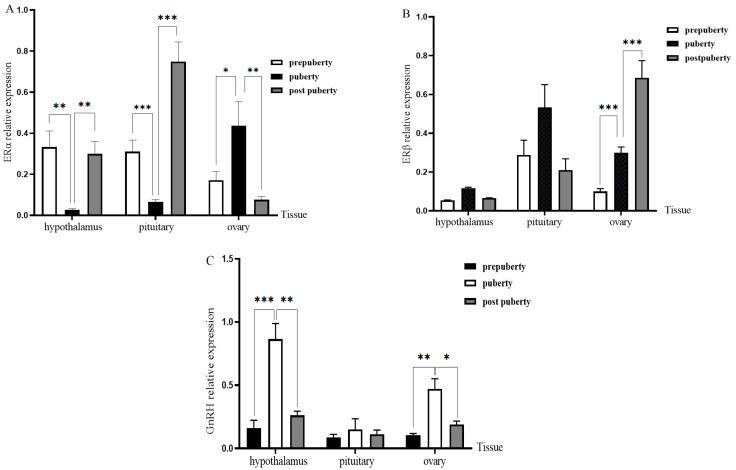
Relative mRNA expression of ERα (**A**), ERβ (**B**), and GnRH (**C**) in the hypothalamus, pituitary, and ovary at different pubertal stages. Data were normalized to ACTB and expressed as fold change vs. prepuberty (set as 1.00). Values are mean ± SEM (*n* = 6). * *p* < 0.05, ** *p* < 0.01, *** *p* < 0.001.

The transcriptional levels of ERα, ERβ, and GnRH in the hypothalamus, pituitary, and ovary were evaluated by quantitative real-time PCR (qPCR) across three pubertal stages (Figure 5). ERα expression in the hypothalamus and pituitary was significantly downregulated at puberty and subsequently increased at the postpubertal stage. Specifically, relative to the prepubertal group (set to 1.00), ERα expression decreased to 0.56 ± 0.11-fold and 0.61 ± 0.09-fold at puberty (*p* = 0.038 and *p* = 0.044), and then increased to 0.94 ± 0.13-fold and 0.89 ± 0.14-fold at postpuberty, respectively. In contrast, in the ovary, ERα expression peaked during puberty (1.85 ± 0.24-fold vs. prepuberty, *p* = 0.041) and subsequently declined to 1.23 ± 0.17-fold in the postpubertal group. ERβ mRNA expression in the ovary exhibited a progressive increase with sexual maturation. Compared to the prepubertal stage (set to 1.00), expression levels significantly increased to 2.47 ± 0.18-fold at puberty and remained elevated at 2.53 ± 0.21-fold postpuberty (*p* = 0.006 and *p* = 0.004). Furthermore, GnRH transcript levels in the hypothalamus also demonstrated a stage-dependent upregulation. Compared with the prepubertal group, GnRH expression was significantly increased to 1.72 ± 0.19-fold at puberty (*p* = 0.034) and further elevated to 2.11 ± 0.23-fold at postpuberty (*p* = 0.018). These findings suggest a coordinated increase in GnRH synthesis alongside ER expression dynamics during the pubertal transition in Duolang sheep.

### 3.5. Expression of ERs and GnRH in Granulosa Cells Stimulated with Different Concentrations of E2

**Figure 6:**
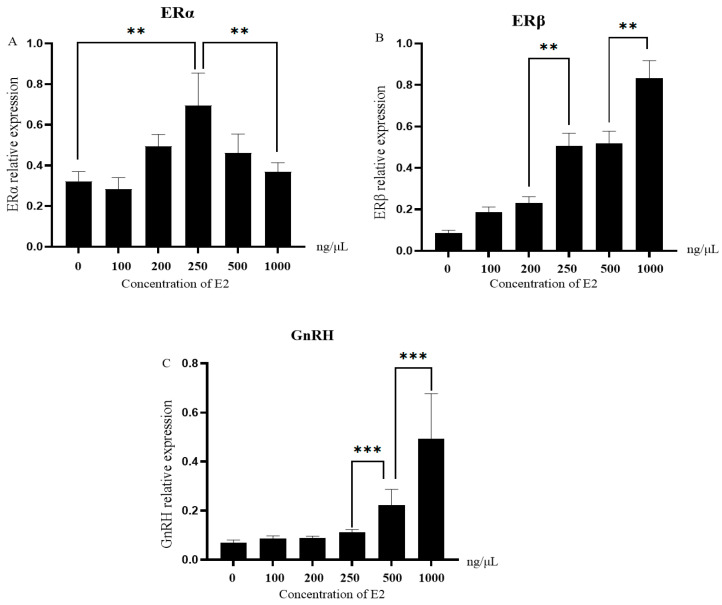
Expression of ERα (**A**), ERβ (**B**), and GnRH (**C**) in granulosa cells treated with different E2 concentrations. Expression was normalized to ACTB and shown as fold change vs. 0 ng/mL (set as 1.00). Data are mean ± SEM (*n* = 3). ** *p* < 0.01,*** *p* < 0.001.

To investigate the dose-dependent response of granulosa cells (GCs) to E2, primary GCs were treated with increasing concentrations of E2 (0, 125, 250, 500, and 1000 ng/mL) and the expression levels of ERα, ERβ, and GnRH were quantified by qPCR (Figure 6). ERα expression initially increased, reaching a peak at 250 ng/mL (2.12 ± 0.23-fold vs. 0 ng/mL, *p* = 0.029), followed by a decline at 1000 ng/mL (1.34 ± 0.17-fold, *p* = 0.041), indicating a biphasic regulatory pattern. In contrast, ERβ expression displayed a dose-dependent elevation across all tested concentrations, rising to 3.11 ± 0.29-fold at 1000 ng/mL compared to the control (*p* = 0.002).

Similarly, GnRH expression in GCs exhibited a significant upregulation with increasing E2 concentrations. Transcript levels increased to 1.58 ± 0.15-fold at 125 ng/mL (*p* = 0.047), 2.34 ± 0.18-fold at 250 ng/mL (*p* = 0.026), 2.85 ± 0.22-fold at 500 ng/mL (*p* = 0.009), and peaked at 3.64 ± 0.31-fold under 1000 ng/mL E2 treatment (*p* = 0.003). These results demonstrate that ERs and GnRH are differentially regulated in GCs in response to escalating estrogen levels, suggesting a potential role in follicular responsiveness to E2 during the peri-pubertal period.

### 3.6. Western Blot Analysis

**Figure 7:**
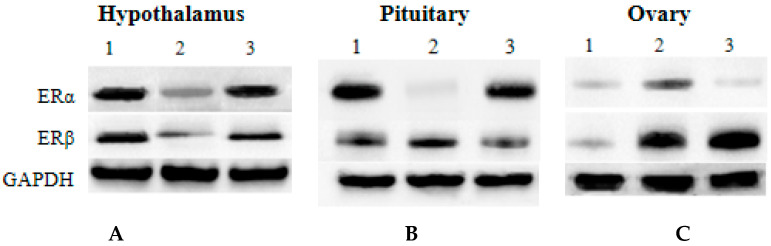
Western blot of ERα and ERβ in hypothalamus, pituitary, and ovary at each stage. GAPDH served as the internal control. (**A**) represents prepuberty, (**B**) represents puberty, (**C**) represents postpuberty.

**Figure 8:**
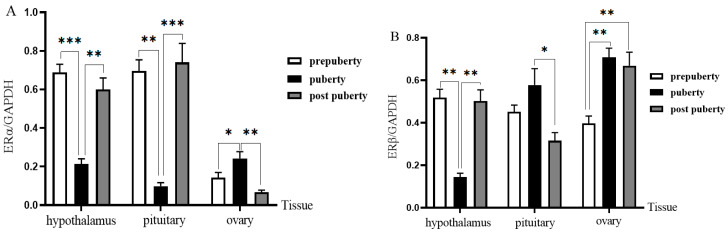
Quantified protein levels of ERα (**A**) and ERβ (**B**) normalized to GAPDH and expressed as fold change vs. prepuberty (set as 1.00). Values are mean ± SEM (*n* = 3). * *p* < 0.05, ** *p* < 0.01, *** *p* < 0.001.

Western blot showed that ERs proteins were expressed in the hypothalamus, pituitary gland and ovaries at different stages (Figure 7 and Figure 8). Western blot confirmed that the levels of ERα protein in the hypothalamus and pituitary gland during puberty were 0.59 ± 0.10 and 0.63 ± 0.12 times before puberty, respectively (1.00 compared with prepuberty; *p* = 0.045 and *p* = 0.039). In contrast, ERα expression in the ovaries peaked during puberty (1.96 ± 0.22-fold, *p* = 0.036). The expression of ERβ in ovaries was significantly higher in adolescence (2.58 ± 0.27 times) and postpuberty (2.62 ± 0.25 times) than in prepuberty (*p* = 0.005 and *p* = 0.004).

### 3.7. ERs Cloning

**Figure 9:**
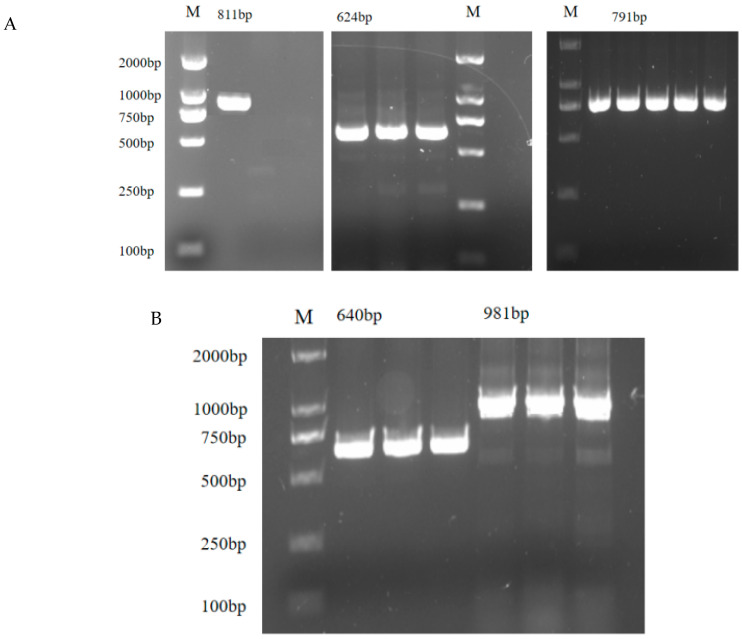
(**A**,**B**) ERs products detected by gel electrophoresis.

The PCR products of ERα and ERβ were detected by 1.5% agarose gel electrophoresis. It could be seen that the target fragments of ERα (Figure 9A) and ERβ (Figure 9B) were consistent with the prediction. BLAST comparison (NCBI BLAST, version 2.14.0) showed that the sequences obtained from clone sequencing were 99.89% and 99.49% homologous to Ovisaries sequences.

**Figure 10:**
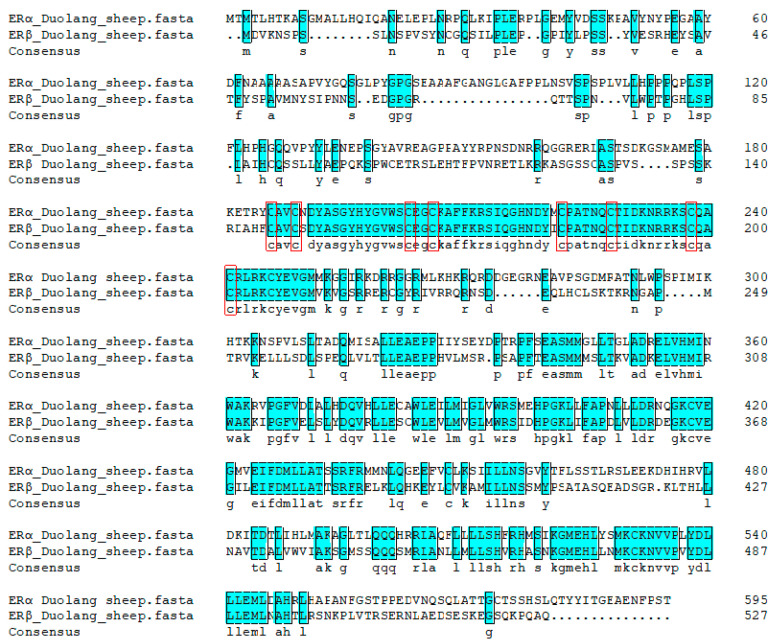
Comparison of ERα and ERβ amino acid sequences of Duolang sheep. The labeled regions were identical sequences of ERα and ERβ. Positions of eight cysteines in ERα and ERβ of Duolang sheep. The red square represents the locations of the eight cysteine residues.

**Figure 11:**
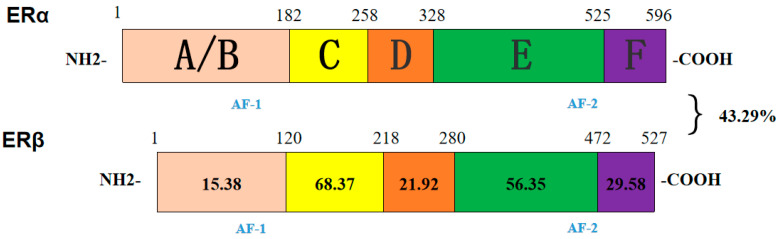
Comparison of homology of ERα and ERβ amino acid sequences in Duolang Sheep. AF-1 and AF-2 can be found in domains A/B and E in their sequences.

The amino acid composition of ERα and ERβ was different (Figure 10), but they were divided into six regions: A/B, C, D, E and F (Figure 11). The homology of ERα and ERβ was 43.29%. C and E had high homology, 68.37% and 56.35%, respectively. And there were eight cysteine residues with the same position in C.

### 3.8. Homologous Sequence Analysis and Evolutionary Tree Construction

**Figure 12:**
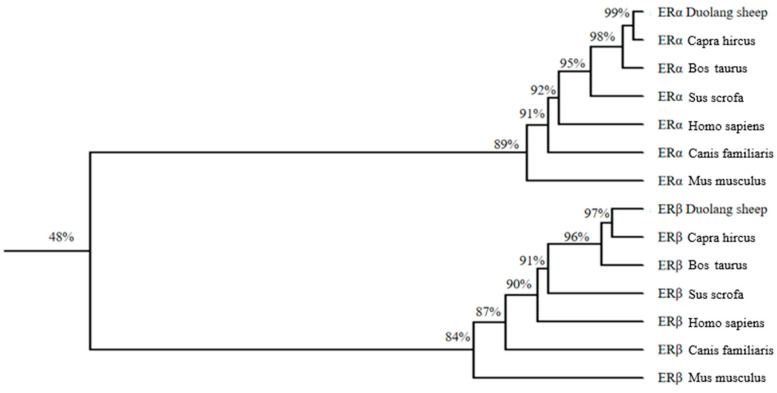
Evolutionary tree construction of ERα and ERβ in different species. Duolang sheep ERs were compared to *Capra hircus* (XP_042109569.1, NP_001272617.1), *Bos taurus* (NP_001001443.1, NP_776476.2), *Sus scrofa* (XP_020938716.1, NP_001001533.1), *Homo sapiens* (NP_000116.2, NP_001035365.1), *Canis familiaris* (XP_038509304.1, XP_038529762.1) and *Mus musculus* (NP_001289460.1, NP_034287.3) and an evolutionary tree was constructed.

ERα and ERβ were compared with Capra hircus, Bos taurus, Sus scrofa, Homo sapiens, Canis familiaris and Mus musculus to construct an evolutionary tree (Figure 12). The evolutionary tree showed that ERα and ERβ belonged to two different proteins. The highest homology of the ERα amino acid sequence of Duolang sheep was 99.9% with goats, but only 89.1% with musmusculus. The highest homology with ERβ was 97.3% with goat and only 84.1% with Homo sapiens.

### 3.9. Bioinformatics Analysis

ExPASy showed that ERα was composed of 596 amino acids, the molecular formula was C_2935_H_4615_N_827_O_857_S_41_, and the molecular weight was 66,513.42. The isoelectric point was 8.11. The instability index was 48.93, indicating that it was an unstable protein. The total hydrophilic index was −0.329, which was hydrophilic protein. ERβ was composed of 527 amino acids with the molecular formula C_2588_H_4145_N_739_O_772_S_39_ and a molecular weight 59,215.21. The isoelectric point was 8.85. The instability index was 62.44, indicating that it was unstable protein. The hydrophilic index was −0.349, belonging to hydrophilic protein. PSORTII was used to analyze the localization of ERα and ERβ in cells. It was found that both ERα and ERβ existed in cells, mainly in the nucleus, accounting for 73.9% and 78.3%, respectively. TMHMM ServerV.2.0 showed that ERα and ERβ had no transmembrane structure.

## 4. Discussion

The findings indicate that E2 plays a crucial role in the onset of puberty in Duolang sheep, with ERs mediating its effects on reproductive tissues. The dynamic changes in E2 levels and ER expression highlight the complex regulatory mechanisms involved in sheep puberty. Further studies are warranted to explore the specific roles of ERα and ERβ in ovarian development and function.

ERα and ERβ sequences are highly conserved. Phylogenetic trees conform to the law of evolution. ERα and ERβ are located in the cell and occupy the largest proportion in the nucleus, which belongs to the nuclear receptor family. Both proteins are hydrophilic and have no transmembrane structure, which is consistent with their transcription pattern.

The structures of ERα and ERβ are highly homologous, both of which have six functional domains A/B, C, D, E, and F [14] Domain A/B are relatively low in conservation and contain the ligand-independent activation function 1(AF-1) transcription functional region, which can combine with transcription factors to regulate target genes [15] Domain C of ERα and ERβ have the same distribution of eight cysteines which control the selection of ERs for target genes [16]. Domain D contains a nuclear localization signal that can influence intracellular region division and post-translational site modification [17]. Domain E is the ligand binding domain, which contains an activation function 2(AF-2) region that can specifically bind E2. Domain F is the carbon terminal binding domain. ERs are the only receptors in this region among all sexual steroid hormone receptors, and their function is poorly understood [18]. ERα and ERβ of DL are highly homologous in the domains C and E, but the domain A of the two receptors is quite different [19]. The amino acids of ERα and ERβ of Duolang sheep have certain homology, the total homology is 42.95%. The similarity of ERα and ERβ in domains C and E is higher than 50%, indicating that ERα and ERβ have similar three-dimensional structures to some extent and play the same role.

E2 pulsated in DL puberty and reached the highest in puberty, suggesting that E2 was involved in the regulation of animal puberty. E2 can regulate the physiological functions of the hypothalamus, pituitary, and ovary through positive and negative feedback. E2, as a steady-state messenger between the hypothalamus and gonad, mainly regulates the biosynthesis and secretion of GnRH neurons. The positive feedback effect of E2 on GnRH is reflected in the increase in GnRH secretion, thus inducing ovulation and other processes. E2 can regulate the transcription of GnRH and the activity of GnRHR promoter. E2 can also mobilize intracellular Ca^2+^, leading to the activation of protein kinase C metabolic pathway, thus regulating GnRH and GnRHR expression. E2 can inhibit GnRH and gonadotropin release through its negative feedback during most of the ovarian cycle. Removing the ovaries disrupts this feedback, leading to elevated gonadotropin levels. The plasma gonadotropin level decreased rapidly when stimulated by a certain dose of exogenous E2. E2 plays the same role in the hypothalamus and pituitary to ensure consistent ovulation timing. At the same time, E2 and progesterone can stimulate the nerve center together, making ewes show restlessness, loss of appetite, and other behaviors during estrus.

However, no obvious behavioral changes were observed in puberty sheep, a phenomenon also observed in other mammals, where progesterone is essential for full expression of estrous behavior [20]. Such as emotional restlessness and random walking. The main reason is sheep show obvious estrus behavior requiring progesterone and E2. However, the luteum did not form during the whole process at the onset of puberty. Therefore, the low level of progesterone in the sheep was manifested as quiet estrus. E2 also has the function of stimulating the development of reproductive organs. After entering puberty, the development rate of reproductive organs is accelerated. The average concentration of E2 in postpuberty was higher than that in prepuberty, suggesting that E2 can stimulate the development of reproductive organs. E2 levels in heifer in postpuberty were higher than in prepuberty, and its common function was to accelerate the growth of the ovary and improve its reproductive function.

E2 must bind to ERs in target organs or target cells to function [21]. Many studies have shown that ERsare expressed in a variety of animal tissues [22]. ERs are expressed in the hypothalamus, pituitary, and ovary, indicating that E2 has different biological functions in different tissues. In other ruminants, developmental ER expression during puberty has been documented. In JiNing grey goats, ovarian ERα and ERβ mRNA decreased from birth to 30 d, then significantly increased at ~60 d when ovulation began, remaining elevated into puberty [23]. In dairy cattle, ERα immunoreactivity in the hypothalamus varies across estrous cycle phases, with strong expression in luteal but low in estrus/metestrus [24]. In the present study, although no formal correlation analysis was conducted, we observed that temporal trends in serum E2 levels roughly paralleled the expression patterns of ERα and ERβ, particularly in ovarian tissue. This suggests a potential regulatory association worth further investigation. Additionally, in pubertal female goats, serum concentrations of estradiol, LH, and FSH sharply rise, accompanied by upregulated ovarian ER-related gene expression [25]. These data indicate ER expression adapts to reproductive stage and estrogen feedback. By contrast, here we chart for the first time the tissue-specific, temporal ERα/ERβ expression in Duolang sheep during puberty, filling a gap in ruminant reproductive endocrinology.

In combination with the mRNA and protein levels of ERα in the hypothalamus and pituitary and the expression trend of GnRH in the three periods in this study, the negative feedback effect of E2 was realized by ERα. After hypothalamus ERα was destroyed, the time of mice entering puberty was significantly earlier. In the pituitary of mice ERα destroyed, the HPG axis was disturbed, leading to the secretion disorder of reproductive hormones [26] and the estrus cycle [27].

In postpuberty, the E2 level of Duolang sheep was higher than that in prepuberty. In immature reproductive organs of mammalian animals, E2 promotes growth through ERβ and ERβ regulates ERα. In our study, we found that the expression of ERβ was predominant in the ovary compared with ERα, suggesting that E2 stimulated ovarian development through ERβ. ERβ mutation cannot combine with E2, resulting in ovarian failure and failure to enter puberty [28]. ERβ was observed in each stage of ovarian development by immunohistochemistry, indicating that ERβ has a regulatory effect on ovarian growth and development.

The expression of ERβ in the ovary began to increase at the onset of puberty, indicating that ERβ can stimulate GCs growth under the action of E2. Current studies on ERβ showed that its function was mainly related to ovarian follicular development [29]. The expression of ERβ increased gradually and the density of GCs increased after 24 h of E2 stimulation, indicating that ERβ can promote the growth and development of GCs. Female ERβ-deficient mice had fewer follicles and impaired follicle maturation, as well as reduced ovulation, suggesting that ERβ also plays an important role in ovulation. With the increase in E2 concentration, the expression of ERβ and GnRH in GCs increased, suggesting that ERβ may regulate GnRH expression directly or indirectly in GCs. GnRH is implicated in ovarian steroidogenesis and the transcription of several genes involved in the process of follicular maturation and ovulation. GnRH was located in GCs before ovulation, and was also detected in the luteum. It has not been determined whether the effect of ERβ on ovulation is direct or by promoting GnRH. When the GCs were stimulated by a low concentration of E2, there was no significant change in each gene.

However, when the concentration of E2 is higher, the genes have significant changes, indicating that only when the concentration of E2 in the ovary reaches a certain level, the ovarian ERα, ERβ, and GnRH can play their roles. Interestingly, the expression of ERα reached its highest level at 250 ng/mL but decreased significantly at 1000 ng/mL, which was similar to that in the hypothalamus. Is ERα in the ovary consistent with the hypothalamus? Recent studies have shown that ERα combined with E2 can significantly promote the growth of ovarian granulosa cell tumors. Our current study is unable to clarify whether it has a negative feedback effect on the ovary and its role in the onset of puberty. which may reflect a tissue-specific feedback regulation mediated by ERα, as previously reported in rodent models [30].

In summary, E2 changed dynamically, suggesting that E2 was involved in the onset of DL puberty. The expression of ERs mRNA and its protein were different in HPO tissues, suggesting that the hypothalamus, pituitary, and ovary were important targets for E2 to regulate puberty. DL GCs under different concentrations of E2 stimulation, ERβ can promote the high expression of GnRH. Therefore, E2 has potential regulatory effects on ovarian development and follicular growth of sheep.

Although this study is mechanistic in nature, the insights gained into the hormonal regulation of puberty in Duolang sheep may have valuable implications for animal production. Understanding how estradiol and estrogen receptors modulate puberty onset and ovarian function could help inform the selection of breeding stock with desirable reproductive traits, identify biomarkers of sexual maturity, and optimize the timing of estrus synchronization protocols. In the long term, such knowledge could support genetic or nutritional strategies to enhance reproductive efficiency, contributing to improved flock fertility, meat production consistency, and overall food security in sheep-farming systems.

## 5. Conclusions

This study demonstrates that estrogen (E2) plays a pivotal role in the onset of puberty in Duolang sheep, with levels peaking during this critical developmental stage. The differential expression patterns of estrogen receptors ERα and ERβ reveal their distinct regulatory functions: ERα mediates negative feedback in the hypothalamus and pituitary, while ERβ promotes ovarian follicular development and enhances GnRH expression in granulosa cells, particularly under high E2 concentrations. These findings underscore the tissue-specific roles of ERs in coordinating reproductive maturation through the hypothalamus-pituitary-ovary axis. The results provide valuable insights into the hormonal mechanisms governing puberty in sheep.

## Figures and Tables

**Table 1:** Primer sequences used for qPCR.

Gene	Forward Primer (5′→3′)	Reverse Primer (5′→3′)	Product Size (bp)
*ERα*	CCCTCCACGATCAAGTCC	AGGTTGGGAGCAAATAGGA	112
*ERβ*	TCTGGTCTGGGTGATTGC	TGTTCCATGCCCTTGTTA	117
*GnRH*	ATTGTTCACCAGTCCCATTC	TTCCTCTGCCCAGTTTCC	83
*ACTB*	CCAACCGTGAGAAGATGACC	CAGAGGCGTACAGGGACAG	96

## Data Availability

The datasets used and analyzed during the current study are available from the corresponding author on reasonable request.

## Abbreviations

The following abbreviations are used in this manuscript:

ERs	Estrogen Receptors
DL	Duolong Sheep
GCs	Granulose Cells
qPCR	Quantitative Polymerase Chain Reaction
RT-PCR	Reverse-Transcription Polymerase Chain Reaction
ERα	Estrogen Receptors Alpha
ERβ	Estrogen Receptors Beta
GnRH	Follicle-Stimulating Hormone
HPO	Hypothalamus–Pituitary–Ovary
FSH	Follicle-Stimulating Hormone
LH	Luteinizing Hormone
DMEM	Dulbecco’s Modified Eagle Medium
PBS	Phosphate-Buffered Saline
RNA	Ribonucleic Acid
GADPH	Glyceraldehyde-3-Phosphate Dehydrogenase (housekeeping gene)
PVDF	Polyvinylidene Fluoride
ELISA	Enzyme-Linked Immunosorbent Assay
SDS-PAGE	Sodium Dodecyl Sulfate-Polyacrylamide Gel Electrophoresis
SEM	Standard Error of the Mean
P	Standard Error of the Mean
E2	17β-Estradiol (Estrogen)
